# Formation Mechanisms of Protein Coronas on Food-Related Nanoparticles: Their Impact on Digestive System and Bioactive Compound Delivery

**DOI:** 10.3390/foods14030512

**Published:** 2025-02-05

**Authors:** Xin Rui, Kai Fu, Hao Wang, Tiange Pan, Wenjun Wang

**Affiliations:** College of Biosystems Engineering and Food Science, National-Local Joint Engineering Laboratory of Intelligent Food Technology and Equipment, Zhejiang University, Hangzhou 310058, China

**Keywords:** protein corona, food nanoparticle, migration, probiotics, food packaging

## Abstract

The rapid development of nanotechnology provides new approaches to manufacturing food-related nanoparticles in various food industries, including food formulation, functional foods, food packaging, and food quality control. Once ingested, nanoparticles will immediately adsorb proteins in the biological fluids, forming a corona around them. Protein coronas alter the properties of nanoparticles, including their toxicity, cellular uptake, and targeting characteristics, by altering the aggregation state. In addition, the conformation and function of proteins and enzymes are also influenced by the formation of protein coronas, affecting the digestion of food products. Since the inevitable application of nanoparticles in food industries and their subsequent digestion, a comprehensive understanding of protein coronas is essential. This systematic review introduces nanoparticles in food and explains the formation of protein coronas, with interactions between proteins and nanoparticles. Furthermore, the potential origin of nanoparticles in food that migrate from packaging materials and their fates in the gastrointestinal tract has been reviewed. Finally, this review explores the possible effects of protein coronas on bioactive compounds, including probiotics and prebiotics. Understanding the formation mechanisms of protein coronas is crucial, as it enables the design of tailored delivery systems to optimize the bioavailability of bioactive compounds.

## 1. Introduction

Nanomaterials, characterized as materials with at least one dimension in the range of 1 to 100 nm, have emerged as an exciting class of materials due to their unique features, including large surface area and mechanical intensity [1]. These nanomaterials exhibit excellent magnetic, electrical, and catalytic properties in contrast to their bulky counterparts [2]. These exceptional benefits contribute to their widespread application in various sectors, including food, medicine, agriculture, environment, and so on [3]. In food industries, nanomaterials show versatile applications. Specifically, they can function as food additives or food supplements to improve the stability, characteristics, and nutrients of foods in food formulation, as nano-carriers to deliver bioactive compounds and enhance their quality and bioavailability in functional foods, as nanofillers to provide antibacterial and antioxidant properties for films and extend the shelf-life of foods in food packaging, or as nano-sensors to detect toxins and pathogens in food quality control [4,5,6,7]. Beyond the food sector, nanomaterials have proven essential in the medicine industry, such as drug delivery, biosensing, and tissue engineering, due to their biocompatibility and controlled release properties [8,9]. Furthermore, nanomaterials also play an important role in the environment sector, particularly in treating contaminants through adsorption, remediation, and membrane filtration [10].

One of the most intriguing aspects of nanomaterials is their interaction with biological molecules when inhaled or ingested [11]. Upon entering the biological environment, nanoparticles interact with biomolecules in biological fluids, especially proteins, leading to a corona around nanoparticles [12]. Although biological fluids contain various biomolecules, such as proteins, lipids, and DNA, the term “protein corona” still dominates in the literature as early studies began with the kinetic of protein association and dissociation on nanoparticles, emphasizing the crucial role of proteins [13]. As a result, this review has focused on the sophisticated formation of protein coronas instead of other biomolecule coronas, such as lipid coronas and polysaccharide coronas [14].

Protein coronas refer to the layers of proteins that adsorb onto the surface of nanoparticles due to the interactions between proteins and nanoparticles, a phenomenon first mentioned by Cedervall and his colleagues [15,16]. This process is highly dynamic because proteins possess different binding affinities towards nanoparticles, which leads to changes in the composition of the protein coronas over time [17]. Initially, proteins with high abundance in the environment tend to adhere to the nanoparticle surface. These proteins typically exhibit weaker binding affinities, but their higher concentrations contribute to rapid adsorption. As the incubation time increases, proteins with stronger affinities gradually replace the initially adsorbed proteins, eventually forming a stable protein corona, which causes the formation of protein coronas to be more sophisticated [18].

The interactions between nanoparticles and proteins are not limited to basic adsorption, which also affects the surface charge and aggregation state of nanoparticles and plays a critical role in how nanoparticles interact with the biological environment [19]. This situation is particularly significant when nanoparticles are used to deliver bioactive compounds. Bioactive compounds are usually extracted and purified from natural materials, with others generated through chemical synthesis, which are gradually replaced by natural ones [20,21]. Bioactive compounds can interact with one or more compounds of living organisms, tissues, or cells to provide beneficial effects, such as antibiotic, antioxidant, and anticancer properties [22]. However, many bioactive compounds, such as polyphenols, probiotics, and vitamins, show low bioavailability due to their poor water dispersity and fast metabolism under physiological conditions [20]. To address the former issues, many nano-carriers are utilized to enhance the solubility and stability of bioactive compounds throughout the gastrointestinal tract [23]. However, nano-carriers belong to nanomaterials, showing the potential to form protein coronas, which affects the interaction of nano-carriers with the biological environment and the subsequent release of encapsulated bioactive compounds [24]. As a result, research is needed to better understand the impact of protein coronas on the bioavailability of bioactive compounds.

Several reviews have already addressed the formation of protein coronas and their effects on various physiological activities, including cellular uptake, cytotoxicity, and immune response [12,25,26]. These articles have contributed to our knowledge of how nanoparticles interact with the biological system. However, a critical gap exists in the existing reviews regarding the specific origins of nanoparticles in food. In particular, limited attention has been paid to their interactions with digestive enzymes, such as pepsin or trypsin. Moreover, the influence of protein corona and even digestive enzyme corona on bioactive compound delivery is insufficient or has focused on polyphenols, ignoring other bioactive compounds [27].

This systematic review has complemented the potential sources of nanoparticles in food, which include bioactive compounds delivered by nano-carriers, such as probiotics and prebiotics, and those migrating from food packaging materials. Specifically, our objective is to provide a comprehensive overview of food-related nanoparticles and their interactions with proteins and enzymes in the gastrointestinal environment. Moreover, we introduce the effects of the resulting protein coronas on their composition and bioavailability of bioactive compounds.

**Figure 1:**
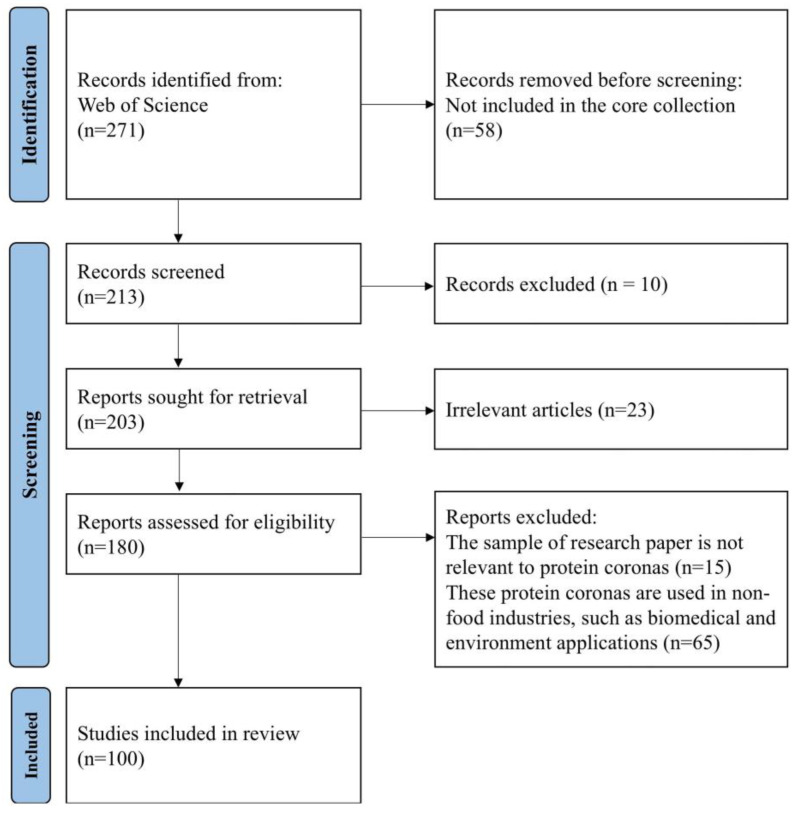
PRISMA flow chart with articles included in the present systematic review.

To conduct this study, the authors followed the PRISMA guidelines for performing a systematic literature search using the Web of Science database, with search dates from its inception to December 2024. The search was conducted using the following keywords: “protein corona OR biomolecular corona OR biocorona” AND “nanoparticle OR nanomaterial OR nanotechnology” AND “food.” Initially, 271 documents were obtained. The first set of exclusion criteria included (1) non-English literature and (2) document types other than reviews and articles. Irrelevant articles focused on eco-corona formed by nanoplastics and biocorona formed by lipids and polysaccharides. Ultimately, 100 articles were selected based on the former criteria. The PRISMA flow chart, which outlines the number of documents and their processing at each stage, is attached to illustrate the literature selection process clearly (Figure 1).

## 2. Nanoparticles in Food

Nanomaterials are widely used in various food industries, and nanoparticles are the most common type of nanomaterial in food. Due to their excellent properties, such as ultra-small size, high specific surface area, abundant functional groups, and good biocompatibility, nanoparticles play a crucial role in food industries, including food formulation and packaging [28]. These nanoparticles can be classified into two categories: endogenous nanoparticles and exogenous nanoparticles [14].

### 2.1. Endogenous Nanoparticles in Food

Endogenous nanoparticles are nano-structures formed in vivo, and during food thermal processing, a new class of these nanoparticles, named fluorescent carbon dots (CDs), are created through complex interactions among food ingredients [29]. Unlike exogenous nanoparticles for specific applications, contact with these food-borne CDs is common in our daily lives [30]. CDs in real food were first observed in caramels in 2012, formed by heating ingredients without water at the early stages of food processing, leading to the creation of carbon nanoparticles [31].

Specifically, the formation process of CDs begins with the pyrolysis of food ingredients, including proteins, carbohydrates, and lipids, and subsequently creates small fluorescent molecules. As the heating continues, short and long fluorescent polymer chains are gradually produced, which undergo further condensation and ultimately form carbon cores. In the final stage, the larger carbon cores break into smaller fragments, forming carbon dots that emit intense fluorescence [30]. Apart from conventional heating methods, microwave heating is a method of inputting energy in food processing, which can also produce CDs. Under intense microwave irradiation, which is a method similar to the daily milk processing method, proteins in milk denature and form aggregates with sugars, followed by in situ carbonization to produce water-soluble carbon dots with an average size of 4 nm [32]. Thus, it is clear that heating or irradiation is essential for forming CDs, which widely exist in condiments, beverages, meat, seafood, and other food items [12].

Fluorescent CDs are typically less than 10 nm in size and mainly contain carbon, oxygen, hydrogen, and sometimes nitrogen. Their surface is rich in functional groups such as amino, hydroxyl, carboxyl, and carbonyl groups [33]. These elements and functional groups impart CDs significant characteristics, including water solubility, chemical stability, biocompatibility, low toxicity, antibacterial activity, and antioxidant properties, which enable CDs to function as nano-carriers for delivering bioactive compounds and biosensors for detecting food-borne microbes, heavy metals, and harmful colorants [34]. These applications will be discussed in the following sections.

### 2.2. Exogenous Nanoparticles in Food

Apart from endogenous nanoparticles generated by food ingredients during processing, exogenous nanoparticles are utilized for various applications in food industries. Exogenous nanoparticles in food refer to nano-structures intentionally introduced into food products through chemical synthesis or extraction from natural materials [29]. These nanoparticles serve a wide range of functions in food industries, such as food additives and food supplements in food formulation, nano-carriers in functional foods, nanofillers in food packaging, and nano-sensors in food quality control [35]. The European Food Safety Authority (EFSA) highlights the importance of assessing the dietary intake of nanomaterials when exposed to the nanomaterial or its degradation products and provides guidelines for the safety assessment of nanomaterials in food [36]. In addition, the United States Food and Drug Administration (USFDA) regulates the use of nanomaterials in food. It requires manufacturers to conduct comprehensive safety assessments for commercially available nanomaterials, including studies on humans and animals, to evaluate their safety under the worst-case conditions [37].

#### 2.2.1. Nanoparticles in Food Formulation

Food formulation is a complex process that involves a careful and selective combination of various kinds and amounts of ingredients. It is designed to meet functional and regulatory requirements while satisfying consumer preferences [38]. Food formulation is particularly crucial for special populations, such as the elderly with dysphagia or diabetes, infants deficient in essential vitamins or minerals, and vegetarians keen on plant-based meals [39,40]. These groups require dietary interventions tailored to supplement their daily nutrient intake [41]. A well-structured food formulation includes essential nutrients such as carbohydrates and proteins, which provide the necessary building blocks for human nutrition and health [42]. In addition to these essential nutrients, the food matrix often incorporates other ingredients for specific functions, such as food additives and supplements.

Food additives are a significant component in food formulation, enhancing the sensory, textural, and rheological properties of food products during processing and storage [43]. A growing trend in food formulation involves utilizing food by-products of both plant and animal origin to pro-duce food additives, such as tartaric acid (E334), which is extracted from tartar and grape pomace and functions as an acidulant to provide an acidic taste [44]. While food additives are primarily used to maintain food quality and safety, food supplements aim to correct nutritional deficiencies or support physiological functions [21]. Both food additives and supplements in their nano forms are increasingly popular, providing a larger surface area that facilitates better absorption in the digestive system [45]. In addition, nano- or micro-structures are applied to enhance the stability and bioavailability of food additives and food supplements, which are discussed in Section 2.2.2 [46]. This section focuses on bare nanoparticles in food formulation.

Specifically, food additives are natural or synthetic substances intentionally added to products to preserve flavor or enhance the appearance, color, taste, and other qualities of food. According to various applications and aims, they can be categorized into different types, containing colorants, preservatives, antioxidants, emulsifiers, gelling agents, and so on [47]. Food-grade additives contain varying amounts of nanoparticles, which increase the efficacy of conventional additives [48]. For example, TiO_2_ nanoparticles account for approximately 36% of food-grade TiO_2_ (E171), a white colorant in chocolate, chewing gums, and other candy products [49]. However, the European Food Safety Authority (EFSA) has raised concerns about the safety of E171 due to its potential genotoxicity and has refused to consider it an additive since 2021, although it remains safe in other countries outside Europe [50]. Iron oxides and hydroxides (E172) are color additives, and the unique forms of iron exempt from certification show excellent dispersity in suspensions and high bioavailability due to iron nanoparticles [51]. Moreover, they can provide various colors in sweets, olives, or cheese rinds, ranging from the black of magnetite (Fe_3_O_4_), the red or orange-brown of hematite (Fe_2_O_3_), to the yellow of FeOOH [52]. In addition, nano-additives are also frequently found in silver (E174), gold (E175), and silicon dioxide (E551), where nanoparticles can even make up more than 50% [53]. More examples and details are listed in Table 1.

Food supplements refer to foodstuffs designed to supplement the regular diet or adjust physiological activities by adding a high concentration of nutrients or other substances, including vitamins, minerals, and polyphenols [54]. Calcium is a human microelement, playing a crucial role in various biological processes, including biomineralization, which contributes to forming and maintaining the health of bones and teeth [55]. Calcium carbonate (CaCO_3_), the most abundant form, is prevalent in providing calcium to supplement its intake. However, bulk CaCO_3_ shows insufficient efficiency in delivering Ca^2+^. As a result, CaCO_3_ nanoparticles are utilized to supplement Ca^2+^, as they improve the absorption of Ca^2+^ through efficient cellular internalization [56]. In addition, Zn deficiency caused by unbalanced dietary issues and diseases, like gastrointestinal tract disorders and cancers, is widespread in children and adults. To address the problem, zinc oxide (ZnO) nanoparticles, a common form of Zn, are frequently applied to supplement Zn [57]. Due to the instability and low bioavailability of some nutrients, which interfere with their applications in food, nano-carrier are an indispensable way to deliver them.

**Table 1:** Common bare nanoparticles in food formulation, including food additives and food supplements.

Food Applications		Type of Nanoparticles	Function	References
Food formulation	Food additive	TiO_2_ nanoparticles	As a white colorant in chocolate, chewing gums, and other candy products	[49]
		Iron oxide and hydroxide nanoparticles	As a food colorant to provide black (Fe_3_O_4_), yellow (FeOOH), and red or orange-brown (Fe_2_O_3_) in candies, olives, and cheese rinds	[52]
		Ag nanoparticles	As a food colorant on the external coating of confectionery to adorn chocolates, confections, and liqueurs	[58]
		Au nanoparticles	As a food colorant to coat or color candies, cake sprinkles, chocolates, and liquors	[59]
		SiO_2_ nanoparticles	As an anti-caking agent to prevent powdered products from clumping; As a stabilizer to ensure the clarity of beer products throughout the manufacturing process	[60]
	Food supplement	Fe_4_(P_2_O_7_)_3_ nanoparticles	As a food fortifier in chocolates and infant cereals to avoid iron deficiency anemia without an unpleasant taste or color	[61]
		FeS nanoparticles	As a food fortifier to improve the absorption of Fe for red blood cells and blood hemoglobulin in broiler chickens	[62]
		CaCO_3_ nanoparticles	As a calcium supplement to enhance cellular uptake efficiency	[56]
		ZnO nanoparticles	As a nutrient fortifier and Zn supplement to prevent zinc deficiencies triggered by illnesses and dietary issues	[57]

#### 2.2.2. Nano-Carriers for Bioactive Compound Delivery in Functional Foods

**Figure 2:**
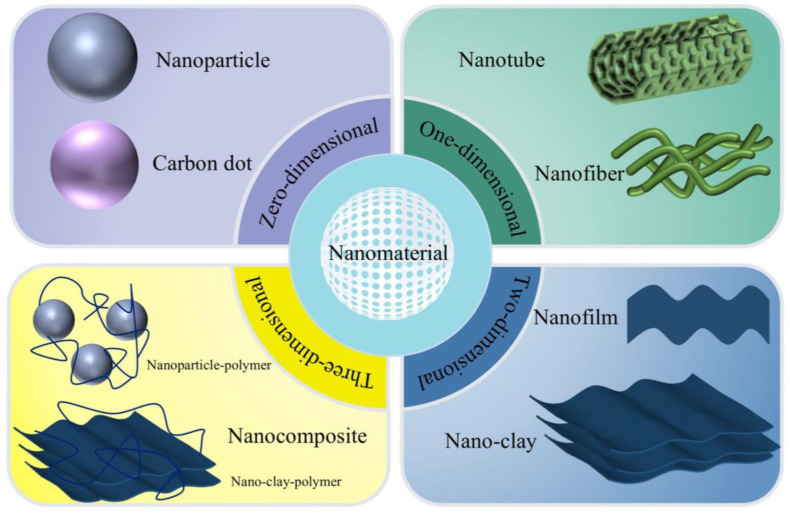
Classification of nano-carriers in the food industry.

Bioactive compounds, encompassing polyphenols, vitamins, and proteins, are considered viable and safer substitutes for synthetic food additives [63]. However, they face challenges, such as limited bioaccessibility, poor solubility, undesirable flavors, and low stability during processing and storage [64]. Innovative delivery systems are employed to address these issues, with nano-carriers emerging as one of the most effective solutions [5]. Nano-carriers are nanomaterials that transport bioactive compounds or drugs, improving their stability and bioavailability [65]. According to the dimensions of various nano-carriers, they can be classified as 0D, 1D, 2D, or 3D nanomaterials (Figure 2). For instance, 0D nano-carriers, which are spherical, include carbon dots and nanoparticles, while 1D nano-carriers containing nanofibers remain nanoscale in diameter, with lengths ranging from nanometer to micron. Meanwhile, nanofilms and nanocomposites are 2D and 3D nano-carriers, respectively [66]. Additionally, nano-carriers can be categorized by their origin as natural biomolecule-based or synthetic polymer-based. Various applications are shown in Table 2.

Nano-carriers based on natural biomolecules are derived from various natural substances such as proteins, polysaccharides, and lipids, each offering unique advantages for specific applications. Among these, protein-based nano-carriers stand out because they possess high encapsulation efficiency for both hydrophilic and hydrophobic nutrients due to their amphiphilic properties. For example, bovine serum albumin nanoparticles have successfully delivered iron and folic acid to fortify stirred functional yogurt for anti-anemic function [67]. However, despite many benefits, nano-carriers manufactured by a single biomolecule are sensitive to external environmental factors, such as digestive enzymes in the gastrointestinal tract. Undoubtedly, the stability and efficacy of nano-carriers present challenges. Therefore, nano-carriers that combine proteins with polysaccharides are advisable, as they prevent proteins from denaturing and aggregating [20]. To illustrate, a blend of gallic acid-modified chitosan and ovalbumin nanoparticles has been developed as an amphiphilic nano-carrier. This system can transport hydrophilic riboflavin and hydrophobic quercetin, improving their stability and slow-release properties [68].

Given hydrophobic bioactive ingredients, such as essential oil, insoluble vitamins, and some phenolic compounds, lipid-based nano-carriers are significant in protecting and controlling the release of these nutrients [69]. Common lipid-based nano-carriers include nano-emulsions, nano-liposomes, solid lipid nanoparticles (SLNs), and nano-structured lipid carriers (NLCs). Nano-emulsions typically encompass three components: oil, water, and surfactant, and three types: oil in water, water in oil, and bi-continuous [70]. For example, astaxanthin, a lipophilic carotenoid and dietary supplement, is light-sensitive. Phospholipid nano-emulsions have effectively encapsulated astaxanthin and protected it from photodegradation [71]. Nano-liposomes have a bilayer membrane formed by phospholipids dispersing and self-assembling in aqueous solution [72]. These nano-liposomes can load essential oil through hydrophobic interaction to preserve its antioxidant and antimicrobial activities [73]. In addition, SLNs, typically a single layer, consist of a crystallized lipid core and a dispersed phase made up of a blend of solid lipids and bioactive compounds [74]. Although this type of lipid-based nano-carrier is conducive to transporting lipophilic bioactive compounds, utilizing a microemulsion dilution method can improve the encapsulation efficiency of hydrophilic nutrients. Ravanfar et al. [75] incorporated anthocyanin into SLNs to protect it from degradation. Since anthocyanin is unstable in the elevated pH of intestine-simulating media, SLNs have been prepared to stabilize this compound, protecting it from harsh conditions of relatively high pH and temperatures along the gastrointestinal tract. Nano-structured lipid carriers (NLCs) represent the next generation of SLNs to overcome the drawbacks of SLNs, such as the premature release of the encapsulated bioactive compounds. Unlike SLNs, NLCs comprise solid and liquid lipids [76]. Hesperetin, a flavanone with restricted aqueous solubility and stability, has been successfully encapsulated into NLCs to achieve sustained release and exhibit excellent in vitro cytotoxicity against glioblastoma cells [77].

In addition, synthetic polymer-based nano-carriers are effectively applied for delivery purposes, although their application is limited in food industries due to their non-biodegradable nature [78]. Whereas vitamin B12 is mainly found in meat and eggs, vitamin B9 is more abundant in vegetables and beans. Consequently, these vitamins are essential for vegetarians and carnivores, respectively. Thus, to improve the stability and oral bioavailability of these vitamins in the simulated gastrointestinal environment, poly(lactic-co-glycolic acid) (PLGA) nanoparticles were utilized to encapsulate vitamin B9 and vitamin B12 [79].

**Table 2:** Various nano-carriers applied in functional foods.

Food Applications	Delivering System	Delivering Nanomaterials	Delivered Nutrients	Function	References
Functional foods	Nanoparticles	Fluorescent nanoparticles from cooked beef broth	Zn^2+^	As a nano-carrier to supplement zinc	[80]
		Chitosan nanoparticles	Selenite	As an alternative to traditional selenium treatment or supplementation; To improve antioxidant activity and achieve the slow release of selenite	[81]
		Gallic acid-modified chitosan and ovalbumin nanoparticles	Riboflavin and quercetin	As an amphiphilic nano-delivery system to transport both hydrophilic riboflavin and hydrophobic quercetin and enhance their stability and slow-release properties; To improve antioxidant activity	[68]
		Bovine serum albumin nanoparticles	Folic acid and Fe	As an anti-anemia supplement to fortify stirred functional yogurt	[67]
	Nanocomposite	Whey protein hydrolysate nanocomposite	Ca	To overcome calcium deficiency and as a precaution against osteoporosis, osteopenia, and arterial hypertension	[82]
		Casein phosphopeptides and chitosan oligosaccharides nanocomposite	Ca	To deliver calcium and improve its bioavailability	[83]
	Micelle	Re-assembled casein micelles	Vitamin D	To enhance the bioavailability and stability of vitamin D and to supply micronutrients in staple foods and beverages	[84]
	Lipid-based nano-carriers	Phospholipid nano-emulsion	Astaxanthin	To supplement astaxanthin by preventing it from photodegradation	[71]
		κ-carrageenan nano-emulsion	Fucoxanthin	To improve fucoxanthin’s low bioaccessibility and poor stability, as well as its possible therapeutic benefits in tumor intervention	[85]
		Chitosan-coated nano-liposome	Caffeine	To prolong caffeine retention time and achieve sustained release in the digestive system	[86]
		Nano-liposome	Cardamom essential oil	To protect the antimicrobial and antioxidant activities of cardamom essential oil after one month, which shows its potential as a food preservative	[73]
		Solid lipid nanoparticles	Gallic acid	To improve the bioavailability of gallic acid in chocolate bars, with better physicochemical characteristics, antioxidant activity, and sensorial properties	[74]
		Solid lipid nanoparticles	Anthocyanin	To stabilize anthocyanin against relatively high pH and temperatures	[75]
		Nano-structured lipid carriers	Hesperetin	To achieve sustained release of hesperetin and exhibit excellent in vitro cytotoxicity against glioblastoma cells	[77]
	Nanotubes	α-lactalbumin nanotubes	Lycopene	To improve the aqueous solubility, stability, and antioxidant activity of lycopene and make it potentially applicable in dairy drinks due to the increased viscosity	[87]
		Halloysite nanotubes	Anthocyanin and phenolic acid	To prolong the release time of anthocyanin and phenolic acids and enhance their nutritional value in yogurt	[88]

#### 2.2.3. Nanoparticles in Food Packaging

Nanomaterials have diverse applications in food packaging, primarily as nanofillers and substrates, like nanofibers. Nanofillers, such as nanoparticles and nano-clays, are widely incorporated in food packaging to improve substrates’ mechanical properties, barrier properties, thermal stability, and optical properties [89]. Nano-clays are particularly popular due to their low cost, high water and gas barrier properties, and thermal stability. Nano-clays are layered mineral silicates arranged in a crystallite structure, including halloysite, montmorillonite, and bentonite [90]. For example, halloysite nano-clay has been successfully integrated into potato starch-based bio-nanocomposite films and has functioned as a nanofiller. As the concentration of nano-clay increases, the films’ water and oxygen barrier and mechanical properties are improved, making it potentially applicable in food packaging [91]. The detailed functions are illustrated in Table 3.

In addition, active packaging encompassing bioactive compounds is a developing and innovative area in the food industry. This packaging system aims at improving food safety and extending shelf-life by releasing bioactive ingredients onto the surface while maintaining packed food quality [92]. These bioactive compounds include essential oil, carbon dots, and nanoparticles, all possessing antioxidant and antibacterial properties. For example, chitosan nanofibers have effectively encapsulated chrysanthemum essential oil through electrospinning and leveraging its antibacterial properties to extend the shelf-life of beef [93]. Similarly, sulfur-functionalized carbon dots are applied to fabricate pectin- and gelatin-based packaging film to enhance their UV and water blocking, as well as antioxidant and antibacterial properties [94].

Unlike active packaging, intelligent packaging can monitor the quality condition and provide this message to consumers or food manufacturers. Typical forms of intelligent packaging include time–temperature indicators, barcodes, and so on [92]. They are designed to quantify whether the target compound exists and mainly compass three types: time–temperature, gas, and freshness indicators [95]. For instance, freshness indicators reflect the deterioration of freshness and mainly cause a color change [92]. ZnO nanoparticles are widely used as antibacterial agents in coatings to extend the shelf-life of fruits and vegetables. The surface of ZnO nanoparticles is abundant in reactive oxygen species, which is generated by the reaction between nanoparticles and water [96]. Thus, a packaging film composed of gelatin, agar, anthocyanin, and ZnO nanoparticles shows improved UV blocking, antioxidant, and antibacterial function. Moreover, this film has successfully functioned as a freshness indicator to reflect the quality of shrimp because of apparent color changes caused by the accumulation of total volatile basic nitrogen [97].

**Table 3:** Applications of nanomaterials in food packaging.

Food Applications		Type of Nanomaterials	Other Substances	Function	References
Food packaging	Normal packaging	Halloysite nano-clay	Potato starch-based bio-nanocomposite films	To reinforce the water and oxygen barrier and mechanical properties of films and make it potentially applicable in food packaging industries	[91]
		Montmorillonite and cellulose nanocrystal	Alginate biopolymer	To decrease the water solubility and water vapor permeability of films, with improving mechanical properties including tensile strength and tensile modulus	[98]
	Active packaging	Poly(ethylene oxide) nanofibers	Tea tree oil	To prolong the shelf-life of beef and protect its sensory quality by improving antibacterial activity	[99]
		Nano-emulsion-based edible coating	Ginger essential oil	Utilizing the strong antibacterial activity of ginger essential oil to increase the shelf-life of chicken breast	[100]
		Chitosan nanofibers	Chrysanthemum essential oil	To prolong the shelf-life of beef by encapsulating chrysanthemum essential oil and taking advantage of its antibacterial activity	[93]
		Chitosan thymol nanoparticles	Quinoa protein and chitosan edible films	Utilizing the antibacterial properties of chitosan thymol nanoparticles to preserve cherry tomatoes and berries with enhanced water vapor barrier capability	[101]
		ZnO nanoparticles	Chitosan and gum arabic edible coating	Utilizing the antimicrobial properties of ZnO nanoparticles to preserve post-harvest bananas with retained nutrients and prolonged shelf-life during storage time	[96]
		Ag nanoparticles	Agar	To enhance thermal stability, water vapor barrier properties, and distinctive antibacterial activity	[102]
		Selenium nanoparticles	Alginate edible coating	To supply selenium and maintain the nutritional value of strawberries with extended shelf-life	[103]
		Sulfur-functionalized turmeric-derived carbon dots	Pectin-/gelatin-based bioactive composite films	To enhance the UV and water blocking and mechanical properties of the film with excellent antioxidant and antibacterial activity	[94]
	Intelligent packaging	Au nanoparticles	Chitosan	As a time–temperature and frozen indicator due to the visual color change; When the temperature rises, more and larger particles will be synthesized to induce the aggregation or agglomeration of Au nanoparticles, which alter colors	[104]
		ZnO nanoparticles	Gelatin- or agar-based color-indicator film and blue anthocyanin	As a freshness indicator to reflect the quality of shrimp because of the total volatile basic nitrogen causing apparent color changes	[97]
		Sugarcane bagasse nanocellulose-based hydrogel	Bromothymol blue or methyl red	As a freshness indicator; When microorganisms generate excess CO_2_, the color of the hydrogel will change because pH-responsive dyes reflect the freshness of the chicken	[105]

#### 2.2.4. Nano-Sensors in Food Quality Control

Nano-sensors are primarily employed to measure the output quantity of biological responses and convert these responses into signals for further interpretation and analysis [106]. These sensors provide quantitative data based on specific biological responses and mainly consist of three types: gas nano-sensors, biosensors, and humidity nano-sensors [95]. Representative examples are shown in Table 4.

Biosensors have widespread applications, such as detecting pathogens, contaminants, and nutrients. Among various nanomaterials used in biosensors, magnetic nanoparticles are particularly common. This nano-sensor type utilizes the interaction between magnetically modified molecules and the other complementary molecules, realizing signal improvement [107]. For example, magnetic Fe/Fe_3_O_4_ nanoparticles have been functioned by antibodies to achieve selective binding between *Listeria monocytogenes* and nanoparticles. When *L. monocytogenes* attaches to the surface of magnetic nanoparticles in a low-field nuclear magnetic resonance, the relaxation time of the water protons in the solution is altered. This change allows for detecting *L. monocytogenes* in milk powder and lettuce [108]. In addition to magnetic nanoparticles, endogenous carbon dots are considered promising nanomaterials for biosensing due to their strong fluorescent intensity and photoinduced redox properties [109]. Carbon dots derived from baked lamb have functioned as a biosensor to detect glucose through an oxidation–reduction reaction. Specifically, glucose oxidase quantitatively converts glucose into hydrogen peroxide (H_2_O_2_), which reacts with Fe^2+^ to form hydroxyl radicals. Once hydroxyl radicals form, they will quench the fluorescence of carbon dots, and the quenching efficiency has a direct relationship with the concentration of glucose [110].

**Table 4:** Examples of nano-sensors used in food quality control and their functional mechanisms.

Food Applications	Type of Nanomaterials	Auxiliary Substances	Function	References
Food quality control	Cellulose nanocrystals	NaCl	As a humidity sensor and a colorimetric indicator; Add NaCl to cellulose nanocrystal suspension to induce a pinkish color in the resulting films, which will shift to a bluish color with elevated humidity	[111]
	Magnetic Fe or Fe_3_O_4_ nanoparticles	Silica and anti-*L. monocytogenes* antibodies	As a biosensor to detect *Listeria monocytogenes* in milk powder and lettuce; Utilizing antibodies to modify magnetic nanoparticles achieves a selective binding between *L. monocytogenes* and nanoparticles, which changes the relaxation time of the solution’s water protons and can be detected by nuclear magnetic resonance	[108]
	Au nanoparticles	Antibody	As a biosensor to detect *Salmonella* Typhimurium; Comparing the average frequency before and after adsorbing *Salmonella* monitored by quartz crystal microbalance and utilizing Au nanoparticles to amplify this signal, which is relevant to the concentration of *Salmonella*	[112]
	Au nanoparticles	EDTA and aptamer	As a biosensor to detect *Staphylococcus aureus* in milk and infant formula; Utilizing the colorimetric method to detect *S. aureus* and show naked-eye change, which is caused by the aggregation of Au nanoparticles	[113]
	Magnetic Fe_3_O_4_ nanoparticles	Polystyrene	As a biosensor to extract Sudan dye from chili powder for quantitative analysis	[114]
	Carbon nanocages in grilled turbot fish	Fe^3+^	As a biosensor to detect the content of ascorbic acid; Because ascorbic acid can reduce Fe^3+^ to Fe^2+^, causing the recovery of fluorescence intensity, which is quenched by carbon nanocages	[115]
	CuO nanoflower	Pralidoxime chloride	As a biosensor to detect and quantify different organophosphate pesticides in cabbage and spinach	[116]
	Fluorescent carbon nanoparticles in baked lamb	Glucose oxidase and Fe^2+^	As a biosensor for glucose detection through an oxidation–reduction reaction; Glucose oxidase can quantitatively convert glucose into H_2_O_2_, which forms hydroxyl radicals with a reaction between H_2_O_2_ and Fe^2+^. When hydroxyl radicals form, they will quench the fluorescence of carbon dots	[110]

## 3. Formation of Protein Corona

Once nanoparticles enter the biological environment, they interact with various biomolecules in body fluids. This interaction contributes to forming a dynamic multilayer coating around nanoparticles, known as the protein corona.

### 3.1. Structure and Formation Mechanism of Protein Corona

Due to the distinct binding affinity and exchange rate of proteins attaching to nanoparticles, protein coronas are generated dynamically with a multilayer structure. This system can be divided into two categories: soft coronas and hard coronas, based on their location [117]. The outside layer, known as the soft protein corona, consists of weakly bound proteins with a shorter lifetime, while the inner layer, or a hard corona, is composed of firmly bound proteins with a longer lifetime [48]. The soft corona consists of proteins that are reversibly adsorbed to the surface of nanoparticles, while the hard corona forms through the irreversible adsorption of proteins [118]. Thus, this progress represents a dynamic screening of proteins, where less stable proteins are easily substituted by those with stronger binding affinity, called the Vroman effect [27]. The effect postulates that the total amount of adsorbed proteins remains roughly constant, with the types of proteins changing.

**Figure 3:**
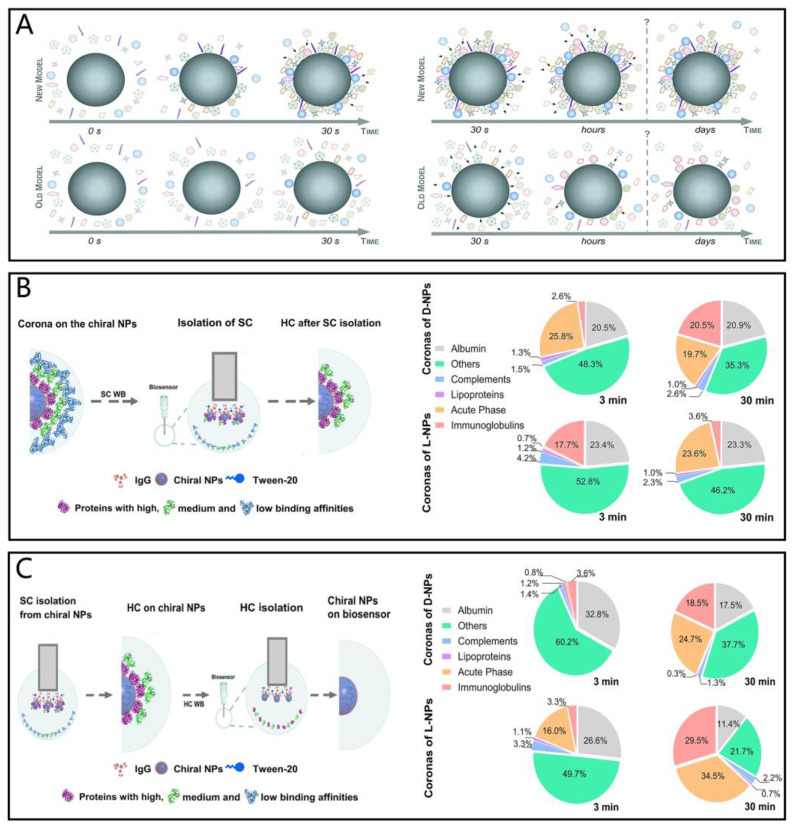
(**A**) Complexity and evolution of the protein corona—the old versus the new model. The old model is fully dependent on the Vroman effect and emphasizes the qualitative change in protein coronas over time, while the new model shows the protein coronas change predominately quantitatively rather than qualitatively [119]. (**B**) Proteomic characterization of time-dependent soft corona components on chiral Cu_2_S nanoparticles. (**C**) Proteomic characterization of time-dependent hard corona components on chiral Cu_2_S nanoparticles [120].

However, due to the complex compositions of protein coronas, which contain more than 200 proteins, the formation mechanism of protein coronas cannot be solely explained by the Vroman effect. Doctor and his colleagues have proposed a new model in which the formation of protein coronas occurs within a short period. They emphasize that the changes in protein coronas over time are predominantly quantitative rather than qualitative (Figure 3A). Additionally, their model suggests that protein coronas adopt multiple core–shell structures or highly ordered “Christmas tree-like” structures, driven by protein–protein interactions, rather than forming a single monolayer [119]. For example, two chiral Cu_2_S nanoparticles form protein coronas with serum proteins. Results imply that the types of adsorbed proteins remain mostly constant in the composition of hard and soft coronas, while the quantities of adsorbed proteins vary greatly (Figure 3B,C) [120]. This further supports the fact that the formation of protein coronas is a dynamic and quantitively changeable process.

In summary, proteins adsorbed on the surface of nanoparticles transform from a loose protein coating to a stable, dense protein coating. The formation mechanism of protein coronas can be explained through several stages. Initially, proteins with a high abundance and rapid diffuse rate adsorb onto the surface of nanoparticles. Two situations exist in the following process:

- If the first adsorbed proteins have low affinities, they will be substituted by those with higher affinities, which exhibit irreversible adsorption onto nanoparticles and form hard coronas.
- On the other hand, once the first proteins successfully bind to the surface of nanoparticles, they tend to retain, and this mechanism continues until the whole surface is covered [118].

Driven by protein–nanoparticle and protein–protein interactions, protein coronas ultimately show multiple layers.

### 3.2. Factors Affecting Protein Corona Formation

**Figure 4:**
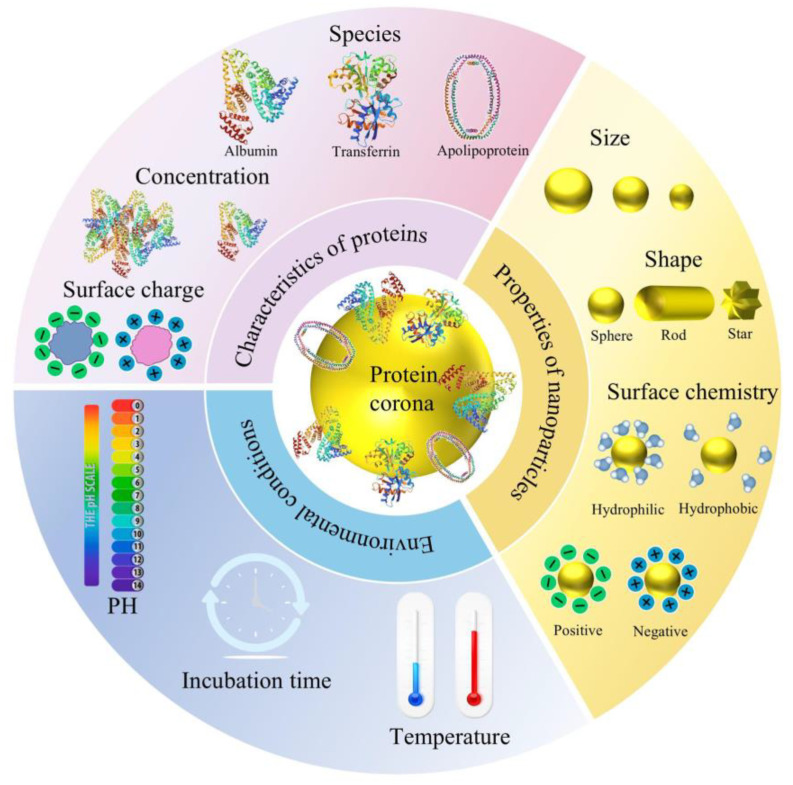
Influencing factors of protein corona formation. Protein structures were taken from the RCSB protein data bank (www.rcsb.org). PDB ID: albumin from human serum: 1BM0; transferrin from human serum: 1D3K; apolipoprotein a-1 from human protein: 1AV1.

Protein coronas are primarily formed through interactions between proteins and nanoparticles, and their formation is influenced by several factors, which can be broadly categorized into three types: physicochemical properties of nanoparticles, characteristics of proteins involved, and environmental conditions (Figure 4). Understanding these influencing factors is crucial for comprehending how protein coronas form.

Various detective and analytical techniques have been developed to study protein coronas. These methods include in situ and ex situ techniques, providing distinct advantages. In situ techniques, such as dynamic light scattering and fluorescence correlation spectroscopy, achieve real-time monitoring of the interactions between nanoparticles and proteins without separating the analyte [121]. However, due to the current limitation in situ methods, more analysis often relies on ex situ techniques, such as liquid chromatography coupled with tandem mass spectrometry and gel electrophoresis, providing deeper insights into the underlying mechanisms of protein corona formation [14]. Several comprehensive reviews have been published on the identification and characterization of protein coronas, focusing on in situ and ex situ techniques [18,27,121]. These reviews provide detailed insights into the various methods and their applications, so readers can refer to these articles for a deeper understanding.

#### 3.2.1. Nanoparticle Properties

Relative properties of nanoparticles include size, shape, surface hydrophobicity, and surface charges, all of which affect the interaction between nanoparticles and biological systems, particularly proteins [122].

The size and shape of nanoparticles determine their curvature, which in turn significantly influences the quantities and types of binding proteins [19]. Larger nanoparticles possess a more extensive interface with smaller curvature to adsorb proteins, with a higher possibility of forming protein coronas. For example, silver (Ag) nanoparticles with an average hydrodynamic size of 20 nm have been shown to bind 30 proteins more than smaller Ag nanoparticles [123]. Similarly, silica (SiO_2_) nanoparticles with a large average size of 71.3 nm preferred to bind proteins, such as prothrombin and thrombospondin-1, while smaller SiO_2_ nanoparticles tended to interact more with lipoprotein clusterin [124]. However, the situation is not always the same. For instance, SiO_2_ nanoparticles with a size of 850 nm exhibit only a single protein band at a molecular weight (MW) of 95 kDa, while smaller SiO_2_ nanoparticles of 60 nm, 100 nm, or 380 nm possess multiple protein bands with a stronger intensity band with MW of 95 kDa [125]. Thus, the impact of nanoparticle size on the quantities of proteins is still ambiguous. In addition to size, the shape of nanoparticles is another crucial factor influencing the amounts of binding proteins. Two mesoporous SiO_2_ nanoparticles share the same size in the γ-dimension and surface chemistry, differing in shapes, with one being a sphere and the other rod. However, SiO_2_ nanoparticles with rod shape have a stronger affinity to proteins from plasma and serum than their spherical counterparts [126].

Surface properties of nanoparticles, such as surface charge and hydrophobicity or hydrophilicity, are significantly related to the interactions with proteins, including electrostatic interaction, van der Waals, hydrogen bonding, π-π interactions, and hydrophobic interactions [14]. The surface charges of nanoparticle surfaces govern the nature of electrostatic interactions and determine whether the interaction between nanoparticles and proteins is attractive or repulsive. Moreover, this affects the composition of protein coronas. For example, gold nanoparticles of approximately 30 nm in size were modified with polyethylene glycol (PEG), carboxylic acid (COOH), and amino (NH_2_) to create nanoparticles with electrically neutral, negative, or positive charges, respectively. Results showed distinct differences in the number and types of binding proteins, with the COOH-coated nanoparticles altering the protein composition to a greater extent than those coated with NH_2_ or PEG [127]. In addition to surface charges, surface hydrophilicity or hydrophobicity represents another influential factor. Hydrophilic surfaces tend to attract proteins through hydrogen bonding. In contrast, hydrophobic surfaces often engage in stronger interactions, such as van der Waals forces, thus interfering with the types and quantities of adsorbing proteins [18]. For instance, apolipoproteins are more likely to bind to hydrophobic nanoparticles, while albumin and fibrinogen preferentially bind to hydrophilic nanoparticles, but hydrophobic surfaces generally adsorb more proteins than hydrophilic surfaces [121].

Given the interrelated nature of these physicochemical properties of nanoparticles, it is sophisticated and challenging to isolate a single property and study its independent influence on binding proteins. As a result, there is still a long way to go, and significant research efforts are needed to elucidate further how these properties impact the formation of protein coronas.

#### 3.2.2. Protein Characteristics

In addition to the physicochemical properties of nanoparticles, the characteristics of proteins, including types, concentration, and charges, can also influence the formation of protein coronas [14].

Due to the sophisticated and intricate nature of in vivo environments, most research on protein coronas uses simulated biological fluids, such as plasma and serum [19]. These fluids vary in the types and quantities of proteins. For instance, SiO_2_ nanoparticles adsorb higher amounts of proteins in human plasma than in human serum. Notably, the former protein coronas exhibit larger sizes and higher levels of coagulation proteins and complement factors [128]. In addition, the concentration of proteins in the incubation media differs, which results in different binding affinity. Proteins at high concentrations can diffuse rapidly and bind to nanoparticles, showing high binding affinity [119].

Moreover, the charge state of proteins is highly related to the electrostatic interaction with nanoparticles. More intensive electrostatic attraction occurs when proteins and nanoparticles exhibit opposite charges on their surfaces, leading to a stable hard corona. For example, within a pH range of 7 to 11, lysozymes were positively charged, while bovine serum albumin (BSA) and anionic SiO_2_ nanoparticles remained negatively charged. Thus, due to more substantial interactions, SiO_2_ nanoparticles preferentially adsorbed more lysozymes than BSA [129]. These findings provide significant insights into protein adsorption in various biological fluids.

#### 3.2.3. Environmental Conditions

Environmental conditions, including pH, temperature, and incubation time, significantly influence the formation and composition of protein coronas. These factors modulate various interactions between nanoparticles and proteins [121].

The surface charges of proteins and nanoparticles are strongly related to the pH of the solution, which further influences the aggregation state of nanoparticles and the electrostatic force between them. Additionally, pH affects protein stability, causing denaturation, unfolding, and self-assembling, interfering with their ability to bind to nanoparticles. Similarly, temperature is a denaturing factor for proteins, which changes their conformation and, as a result, affects the formation of protein coronas. For example, when the pH increased from 4.9 to 8.9, the protein coronas formed by Ag nanoparticles underwent a noticeable shift in composition, with the amounts of 44 proteins increasing while 33 proteins were displaced and decreased. Similarly, as the temperature varied from 4 to 47 °C, 18 proteins increased in abundance, with 48 proteins reduced [130].

In addition, due to the dynamic process of forming protein coronas, incubation time is a crucial factor influencing their composition. For example, when iron oxide nanoparticles were incubated in 100% fetal bovine serum for 24 h, the concentration of the protein corona increased by 59% [131].

## 4. Effects of Protein Corona in Gastrointestinal Tract

Nanomaterials have extensive applications in the food, pharmaceutical, and environmental sectors [132]. In food industries, these materials, which are ultimately ingested and digested by the human body, can be categorized into three types:

- Directly added food ingredients to improve the sensory properties or nutrients of food, including food additives and supplements;
- Unintentionally migrated nanomaterials, originally introduced into food packaging to improve the mechanical properties of films or coatings and extend the shelf-life of food;
- Intentionally incorporated nanomaterials into active packaging to achieve controlled release onto the food surface, including various bioactive compounds with significant antioxidant and antibacterial properties.

Once ingested, these nanoparticles enter the gastrointestinal tract and encounter varying conditions in the mouth, esophagus, stomach, small intestine, and colon [133]. Along this journey, they interact with proteins in biological fluids, forming protein corona, which alters their digestion rate and influences physiological processes.

### 4.1. Migration of Nanoparticles from Packaging to Food

This part focuses on the second category of nanomaterials: those unintentionally migrated from food packaging. Nanoparticles have become a significant component in modern food packaging, particularly in active and intelligent packaging systems. These nanoparticles function as nanofillers or substrates, improving their mechanical properties, and providing antioxidant and antibacterial characteristics to polymer films or coatings. However, despite these advantages, the migration of nanoparticles into food presents a potential threat, exposing the human diet to nanomaterials, which is usually neglected. Thus, understanding the action dynamics of nanoparticle migration is significant, as it provides insights into the migration mechanisms and their potential effects on food safety [134].

Nanoparticles can migrate in two directions simultaneously, from the foodstuff into the packaging material and from the packaging into the food. In the former case, volatile compounds may transfer from the food matrix to packaging through diffusion or evaporation, desorption, and adsorption. This process is strongly related to the principles of intelligent food packaging [135]. In the latter scenario, various nanomaterials, such as metal and metal oxide nanoparticles, nano-clay, and carbon nanotube, may transform into food through different mechanisms [136,137,138].

When assessing the migration of nanoparticles, food simulants are indispensable and the most convenient option for analyzing how the migrants behave in different food matrices. Their physicochemical properties are similar to those of foods and are authorized to be used in migration tests, which simplify the identification and quantification of the resulting migration compounds [139].

Metal and metal oxide nanoparticles are common nanomaterials that migrate into foodstuff primarily as dissolved ions, including Ag, Cu, and ZnO nanoparticles. For example, Ag nanoparticles, when exposed to oxidative conditions, can be oxidized to release Ag^+^, which then diffuses into the food matrix driven by a concentration gradient [140]. The migration rate of these nanoparticles is influenced by various factors, especially those interfering with oxidation reactions, including the concentration, size, shape of nanoparticles, temperature, and oxygen availability. In addition, food matrices, including real food or food simulants, can also impact the extent of nanoparticle release [141]. For instance, Cu nanoparticles were incorporated into low-density polyethylene films, and their migration was assessed under varying conditions. The results revealed that increasing the concentration of Cu nanoparticles, exposure time, and temperature led to a higher migration rate. Moreover, the migration rate of Cu nanoparticles was notably reduced in 10% ethanol than that in 3% acetic acid [142].

In conclusion, the migration of nanoparticles from food packaging presents a complex challenge that needs further research.

### 4.2. Influence of Protein Corona on the Gastrointestinal Fate

Based on the knowledge of nanoparticle migration from food packaging into food, it is essential to understand the subsequent effects once they are ingested along the gastrointestinal tract.

#### 4.2.1. Behavior of Nanoparticles in the Digestive Tract

Upon digestion, food enters the mouth, which is mechanically broken down by chewing and mixed with saliva. Saliva contains immunocompetent compounds and digestive enzymes like amylase. Then, the slightly digested food moves down the esophagus and enters the stomach with highly acidic gastric fluids and digestive enzymes. The resulting chyme is further digested and absorbed in the small intestine with bile, other digestive enzymes, and electrolytes. After this process, undigested food will be delivered into the large intestine and excreted outside [26]. Due to the different pH and ionic strength in the different digestion stages, the aggregation state of nanoparticles will be influenced. For example, the low pH and high electrolyte concentration induce SiO2 nanoparticles to aggregate into larger micro-sized particles in the stomach’s highly acidic environment. However, when digested through the small intestine with a pH of 7, SiO_2_ nanoparticles reappeared in more quantities than in the mouth digestion stage [143].

#### 4.2.2. Impact of Protein Corona on Nanoparticle Properties

As nanoparticles enter the body, they interact with various proteins, which bind to their surfaces due to the high surface energy of the nanoparticles. This interaction generates layers around nanoparticles, forming protein coronas. These protein layers significantly alter the nanoparticles’ physicochemical properties, such as aggregation state and surface chemistry [14]. Due to the adsorption of proteins, nanoparticles’ tendency to aggregate is altered by influencing the interactions between nanoparticles, and this effect varies for different types of proteins [48]. For example, IgG can promote the aggregation of anionic nanoparticles by enhancing interparticle distance and reducing electrostatic repulsion of nanoparticles, while albumin has no such effect [144].

Moreover, the biological behavior of nanoparticles is affected by the formation of protein coronas, including their toxicity, cellular uptake, and targeting characteristics [12]. Specifically, protein coronas may reduce nanoparticle toxicity by serving as a buffer layer and protecting the gastrointestinal epithelia from direct contact with nanoparticles [145]. For instance, when Au nanoparticles are incubated with fetal bovine serum, their aggregation tendency reduces, leading to decreased toxicity, which shows the opposite results when incubated with serum-free media [146]. However, it should be noted that in some cases, protein coronas may enhance nanoparticle toxicity. Human serum albumin forms protein coronas around Au nanoparticles, with increased toxicity of nanoparticles through disrupting cell membranes [147]. Therefore, the impact of protein coronas on nanoparticle toxicity can vary, potentially mitigating or enhancing its toxicity [14].

Regarding cellular uptake, it is evident that the formation of protein coronas influences this property of nanoparticles, which is highly dependent on the size of nanoparticles, with larger nanoparticles (>70 nm) showing a lower uptake rate [121]. For instance, cells take up SiO_2_ nanoparticles forming protein coronas in serum media less than in serum-free media, where nanoparticles show a stronger adhesion to cell membranes. This difference is relevant to the size of protein coverage [148]. In addition, the formation of protein coronas can impair the targeting efficiency of nanoparticles due to the blocking effect of proteins, which is discussed in Section 5 [122].

#### 4.2.3. Impact of Protein Corona on Proteins, Including Digestive Enzymes

Additionally, the interaction between nanoparticles and proteins can alter the proteins’ structure and function. For example, when human serum albumin binds to food-derived carbon dots, the secondary structure changes, decreasing α-helix contents [149]. Moreover, proteins constituting the hard inner layer of protein coronas underwent higher degrees of conformational changes than those in the outside layer [150].

Digestive enzymes, such as α-amylase, lipase, pepsin, and trypsin, are crucial components in the gastrointestinal tract and play significant roles in digestion. Like common biological proteins such as albumin, fibrinogen, and transferrin, these enzymes interact with nanoparticles, forming the digestive enzyme coronas [42,151]. Due to the different affinity between digestive enzymes and nanoparticles, the formative coronas show differentiated characteristics. For example, pepsin and α-amylase have a higher affinity to polystyrene nanoparticles than trypsin, showing more adsorbed digestive enzymes after three centrifugal washing steps to obtain hard coronas [152].

During the formation of the digestive enzyme coronas, the conformation of enzymes has changed, particularly in their secondary and tertiary structures. For example, the interaction between TiO_2_ nanoparticles and trypsin, involving hydrogen bonding and van der Waals force, leads to a decrease in the β-sheet content and an increase in the random coil content [153]. Structural alteration is also observed when resistance starch nanoparticles and pepsin or trypsin form the digestive enzyme coronas. According to the CD spectrum analysis, the α-helix content of trypsin decreases by 3.2%. In addition, the formation of the digestive enzyme coronas has induced fluorescence quenching of digestive enzymes, which shows its impact on tertiary structures [154].

Furthermore, these structural changes can influence the activities and functions of the digestive enzymes after forming the digestive enzyme coronas. The possible influencing mechanisms are related to the unfolded structure caused by the reduction in α-helix and β-sheet contents, the damaged β-hairpin loop aimed at protecting the catalytic sites, and the altered quantities of active sites which are occupied or exposed by nanoparticles [145]. The consequences of digestive enzyme corona formation on the activities of enzymes are complex: negative or positive. For example, when chitin nanowhiskers and α-amylase form a corona through hydrophobic interactions, the secondary structure of α-amylase changes, shifting from α-helix to β-sheet. Moreover, when the concentration of chitin nanowhiskers is 2.5 mg/mL, the α-amylase activity improves by approximately 22% [155]. In addition, the activity of α-amylase and amylopsin is enhanced by 1.4 and 1.6 times due to their interactions with edible dock protein nanoparticles [156]. However, when carbon dots extracted from roasted fish interact with digestive proteases, like pepsin and trypsin, the enzymes undergo conformational changes, which reduces their ability to digest casein [157].

In addition to interacting with digestive enzymes, nanoparticles can interact with gastrointestinal microorganisms, including probiotics and pathogens, leading to an imbalance of gastrointestinal flora [158].

Overall, nanoparticles and proteins form protein coronas in the gastrointestinal tract, which influences the physicochemical properties of nanoparticles, the structure and activity of digestive enzymes, and the balance of gastrointestinal microorganisms.

## 5. Effects of Protein Corona on Bioactive Compound Delivery

Most bioactive compounds possess various beneficial properties, including antioxidant, antibacterial, and anti-inflammatory effects, which prevent and treat several diseases, such as cancer and inflammation [20]. However, the oral delivery of direct bioactive compounds is limited by their poor bioavailability, which refers to the proportion of the compound that reaches the systemic circulation in an active form compared to the total amount ingested [133]. Thus, nano-carriers are applied to enhance the digestibility and stability of bioactive substances.

### 5.1. Common Bioactive Compounds Delivered by Nano-Carriers

In addition to bioactive compounds mentioned in Section 2, such as essential oils, polyphenols, insoluble vitamins, and minerals, this section highlights frequently neglected substances, including probiotics and prebiotics.

Probiotics are live microbes that provide health benefits to the host when consumed in adequate amounts. The most common probiotics are gut microorganisms, such as *Lactobacillus* spp. and *Bifidobacterium* spp. [159]. These probiotics perform significant functions, including inhibiting the overgrowth of pathogens, enhancing the epithelial barrier, and inhibiting illness processes [160]. However, the harsh conditions of the gastrointestinal tract, including acidic pH and digestive enzymes, make it difficult for many probiotic strains to survive. Thus, utilizing nanomaterials to encapsulate probiotics is an effective method to improve stability and bioavailability. Nanofibers manufactured through electrospinning are among the most prevalent nano-carriers [161]. For example, electrospinning technology combines corn starch and sodium alginate to fabricate nanofiber mats. These nanofiber mats encapsulate probiotics, including *Lactobacillus acidophilus*, *Lactobacillus rhamnosus*, *Bifidobacterium bifidum*, and *Bifidobacterium animalis*, to improve their survival in simulated gastrointestinal fluids [162]. In addition, chitosan-based nanoparticles have successfully delivered probiotics, and the system exhibits significant anticancer effects in HeLa cell lines [163].

Prebiotics are typically dietary fibers, primarily oligosaccharides, which are selectively utilized by host microorganisms. They provide various health benefits by modifying host microbiota composition [164]. Specifically, prebiotics can maintain gut health, enhance immune defense, and may also function as food additives and antioxidants [165]. An ideal prebiotic should resist the low pH of the stomach, bile salts, and digestive enzymes in the intestine to avoid premature absorption in the upper gastrointestinal tract. Lastly, it should be easily fermented by the probiotics in the colon [166]. For example, phthalic anhydride and dextran were connected to form a prebiotic. Probiotics internalize the resulting dextran nanoparticles and produce antimicrobial peptides, which could be utilized to prevent the infection of pathogens [167]. Moreover, combining prebiotics and probiotics has been shown to enhance the survival and colonization of probiotics [164]. Therefore, co-encapsulation of probiotics with prebiotics, such as insulin, resistant starch, and polydextrose, offers a promising approach to improve oral delivery and targeted release of probiotics [168].

### 5.2. Influence of Protein Corona on Bioactive Compound Delivery

Various nano-carriers are utilized to deliver bioactive substances, including polyphenols, probiotics, and essential oils. After ingestion, these carriers tend to form protein coronas. As a result, analyzing the influence of protein corona on the delivery of bioactive compounds is crucial.

Throughout the gastrointestinal tract, nanoparticles carrying bioactive compounds become covered with layers of proteins, which interfere with the release and absorption of these compounds. Several possible mechanisms affecting the bioavailability of bioactive compounds are as follows:

- Proteins binding to nano-carriers may protect nano-carriers from breaking down in the gastrointestinal tract, which affects the targeted release of bioactive compounds, such as probiotics intended to function in the colon;
- Digestive enzymes can attach to nano-carriers and form digestive enzyme coronas, which may influence the activities of enzymes by veiling the active sites. This effect potentially interferes with the availability of bioactive substances, particularly if the nano-carrier is based on proteins or carbohydrates;
- Some nano-carriers may resist digestion, which leads to excreting from the digestive system or triggering an immune clearance response and, as a result, prevents the release of bioactive compounds [169].

In conclusion, the formation of protein coronas significantly impacts the availability of bioactive compounds. For example, banana resistant starch nanoparticles have encapsulated epigallocatechin gallate (EGCG), which further interacts with digestive enzymes, forming digestive enzyme coronas. However, the release rate of EGCG has become slower and decreased [154]. Similarly, zein and β-cyclodextrin have been combined to manufacture nanoparticles, which are utilized to deliver curcumin in in vitro simulated digestion. Results show that during the digestion process, digestive enzyme coronas are shown after digestion, which may influence polyphenol availability [170]. However, corresponding research on the interaction between nano-carriers loading bioactive compounds and proteins in biological fluids is insufficient and mainly focuses on polyphenols. Thus, more efforts should be made to research the impact of protein coronas on the availability of bioactive compounds, not limited to polyphenols, but also including other active components, such as probiotics and probiotics.

## 6. Potential Strategies for Modulating Protein Corona Formation

Protein corona formation significantly affects the aggregation state of nanoparticles and the conformation and activity of proteins, as well as the bioavailability of bioactive compounds delivered by nano-carriers. Thus, nanoparticles should be designed carefully to reduce adsorbing proteins and even modulate the formation of protein coronas [171].

The mechanism of protein corona formation is that once nanoparticles enter the body, they form a mixed and unstable colloid system with plasma proteins. In the gastrointestinal tract, nanoparticles characterized by a primarily hydrophobic core easily bind with plasma proteins. To prevent their interactions, we densely coat nanoparticles with hydrophilic and intensely hydrated polymers. This protective effect arises from a combination of a bound water layer and entropic shielding [172]. Various hydrophilic neutral polymeric coatings, such as polyethylene glycol (PEG) and carboxymethyl dextran, have been utilized on chitosan nanoparticles to prevent the formation of protein coronas [173].

In addition, researchers have explored the function of ligands to reduce the formation of coronas [171]. For example, nanoscale metal–organic frameworks have been modified with amino or carboxyl groups, forming protein coronas with bovine serum albumin. Results show that nanoparticles modified with amino groups decrease the binding affinity and the number of proteins, while those modified with carboxyl groups have the opposite effect [174].

In addition, many influencing factors have been discussed in Section 3, including the physicochemical properties of nanoparticles, the types and concentrations of biological proteins, and various environmental conditions. Among them, hydrophobicity is one of the most significant factors that can regulate the formation of protein coronas. Four gold nanoparticles were synthesized with different hydrophobicity ranging from −2.6 to 2.4. According to the experiments, it is clear that hydrophobic nanoparticles adsorbed 2.1 times as many proteins as hydrophilic ones [175]. However, due to the inner connection between the influencing factors, it is not easy to establish a clear relationship between these factors and the formation of protein coronas.

In summary, nanoparticles must be designed with specific strategies to reduce or control protein adsorption, such as using hydrophilic coatings or modifying surface ligands. These strategies contribute to maintaining the target property of nanoparticles and increasing the bioavailability of bioactive compounds.

## 7. Conclusions and Prospects

This review summarizes multiple food-related nanoparticles, including nano-additives and nano-supplements in food formulation, such as TiO_2_ nanoparticles and CaCO_3_ nanoparticles, and nano-carriers in functional foods, like nanotubes and various lipid-based nano-carriers. It also covers nanofillers in food packaging, including ZnO nanoparticles and Ag nanoparticles, and nano-sensors in food quality control, such as Fe_3_O_4_ nanoparticles. Most of them can be ingested and interact with proteins in biological fluids, forming protein coronas. This review then explores the formation of protein coronas, their potential impacts on the gastrointestinal tract, and the bioavailability of bioactive compounds. Moreover, we discuss possible strategies for designing nanoparticles to modulate protein corona formation.

However, due to the potential toxicity of nanoparticles and the adverse effects of protein coronas formation, such as altering the conformation and functions of proteins and digestive enzymes and intervening in the bioavailability of bioactive compounds, food regulations need to pay attention to the use of nanoparticles and nanotechnology in food industries. The following recommendations are proposed:

- In food formulation, regulations should establish strict limits on the presence of nanoparticles in food additives and supplements. It is insufficient to solely regulate the maximum dosage of these substances, as their nano forms exist and present risks to human health.
- In functional foods, the safety of nano-carriers delivering nutrients must be thoroughly evaluated. These carriers must be made from materials that are “generally recognized as safe”. Legislators should establish methods to assess the toxicity of nano-carriers, particularly considering their potential for accumulating in humans over time.
- In food packaging, films containing nanofillers and substrates pose a risk of migration from the packaging materials to the foodstuff. To ensure the safety of consumers, it is essential for legislation to establish reliable and standardized methods to quantify the overall migrated nano-sized components.

Additionally, the formation of protein coronas is a highly dynamic process, influenced by various factors of their compositions, such as the physicochemical properties of nanoparticles and the concentration of proteins. Given the complex interactions between these factors, it is challenging to isolate the individual impact on forming protein coronas. As a result, there is a need to develop a standard and comprehensive evaluation method to gain a deeper understanding of the real effects of influencing factors on protein corona formation to achieve the controlled formation of the protein coronas.

Finally, because of the sophisticated formation of protein coronas, relying solely on in vitro models to simulate the actual gastrointestinal tract is inadequate. Thus, advanced in situ techniques are needed to monitor and analyze the authentic interplay between nanoparticles and proteins.

## Data Availability

No new data were created or analyzed in this study.
